# Clinical outcomes of two-stage revision for chronic periprosthetic joint infection of the knee: culture-negative versus culture-positive

**DOI:** 10.1186/s43019-021-00112-4

**Published:** 2021-09-03

**Authors:** Chang-Wan Kim, Chang-Rack Lee, Dae-Hyun Park, Doo-Yeol Kim, Jeong-Woo Kim

**Affiliations:** 1 Department of Orthopedic Surgery, Inje University Busan Paik Hospital, 75, Bokji-ro, Busanjin-gu, Busan, 47392 Republic of South Korea; 2 Department of Orthopedic Surgery, Good Samsun Hospital, 326, Gaya-Daero, Sasang-gu, 47007 Busan, Republic of South Korea

**Keywords:** Knee, Arthroplasty, Periprosthetic joint infection, Diagnosis, Treatment outcome

## Abstract

**Background:**

The effect of negative culture on the treatment outcomes of chronic periprosthetic joint infection (PJI) is still controversial. The purpose of this study is to evaluate whether the outcomes of two-stage revision in culture-negative chronic PJI differ from those in culture-positive PJI.

**Methods:**

The patients who received two-stage revisions due to chronic PJI during the period between 2007 and 2017 were retrospectively reviewed. The culture-negative and culture-positive PJI group included 57 cases and 79 cases, respectively. The demographic data, as well as reoperation, mortality, reinfection, and failure rates of each group were evaluated.

**Results:**

There was a significant difference in reoperation rate between the two groups for the period from the first-stage surgery to the second-stage revision arthroplasty (*p* = 0.045). The reoperation rate of the culture-positive group was 25.3% (20/79) whereas that of the culture-negative group was 10.5% (6/57). Among the 136 PJI cases, 97 cases (71.3%) received reimplantation surgery (culture-negative group, 43 cases; culture-positive group, 54 cases). No significant difference was noted between the culture-negative and culture-positive groups with respect to the number of cases that did not undergo reimplantation surgery and the reoperation, reinfection, mortality, and failure rates after two-stage surgery (*p* > 0.05, all parameters).

**Conclusions:**

The culture outcome had no significant effect on the outcome of the two-stage revision in patients with chronic PJI. The reoperation rate after first-stage surgery was significantly higher in the culture-positive group, but the overall failure rate did not significantly differ in both the groups. The presence of a negative culture might be a good prognostic factor for chronic PJI.

## Background

Periprosthetic joint infection (PJI) is one of the most serious complications that can occur after total knee arthroplasty (TKA) and is known to be one of the major causes of early failure [1–3]. However, diagnosing PJI is a challenge. In some studies, isolation of the infecting organism failed in more than 40% of the PJI cases [4–6]. A negative culture can delay the diagnosis, making it difficult to determine an appropriate treatment method [6]. Hence, multiple groups, including the Musculoskeletal Infection Society (MSIS), have proposed a diagnostic guideline for PJI. With a series of updates, various serum or joint-fluid biomarkers have been included in the diagnostic criteria [7–11]. However, the detection of a causative organism based on the culture method is still believed to be an important factor in diagnosis.

Among the many treatment options, two-stage revision is the standard treatment of choice for chronic PJI [2, 12]. In addition to surgery, the use of appropriate antibiotics is also important in infection control. Appropriate antibiotics are determined based on the causative agent in culture-positive PJI; however, empirical antibiotics, which are potentially insensitive to the infecting organism, are generally used in culture-negative PJI. Theoretically, it is believed that the use of inappropriate antibiotics increases failure rates in eradicating infection.

The effect of a negative culture on the treatment outcomes of PJI is still controversial. Several published studies have reported that culture-negative PJI and culture-positive PJI show similar clinical outcomes [5, 13–18]. Some authors have reported that reinfection risk was higher in culture-negative PJI than in culture-positive PJI [19]. However, some studies have reported that culture-negative PJI produced better outcomes than culture-positive PJI after the treatment [20]. Further research is required as there are only a few studies that have investigated culture-negative PJI, varying in type of PJI and surgical method.

The purpose of this study is to evaluate whether the outcomes of a two-stage revision in culture-negative chronic PJI differs from those in culture-positive PJI. We hypothesized that the outcomes after the two-stage revisions will not differ between culture-negative PJI and culture-positive PJI.

## Materials and methods

### Patients

This study was approved by our institutional review board. The patients who received surgical treatment due to periprosthetic knee joint infection between 2007 and 2017 were retrospectively reviewed. The inclusion criteria for this study were as follows: (1) patients who received primary or revision TKA in our institution or other institutions/hospitals; (2) patients diagnosed with chronic PJI; and (3) patients who received two-stage revision using antibiotic-impregnated cement spacers. The exclusion criteria were as follows: (1) acute postoperative or acute hematogenous PJI within 1 month from the occurrence of a symptom and (2) patients who did not receive two-stage revision. Because our institution started implementing the electronic medical record system in 2009, among the patients who were diagnosed with PJI or received surgery due to PJI before 2009, those whose data could not be obtained due to medical record disposal were excluded from this study.

The diagnosis of PJI was based on the diagnostic criteria proposed by the MSIS [9]. Periprosthetic joint infection diagnosis is characterized by one of the major criteria or three of the five minor criteria. The major criteria include: (1) two positive periprosthetic cultures with phenotypically identical organisms and (2) a sinus tract communicating with the joint, whereas the minor criteria include: (1) increased serum erythrocyte sedimentation rate (ESR) and C-reactive protein (CRP) (ESR > 30 mm/h and CRP > 10 mg/L), (2) increased synovial-fluid white blood cell (WBC) count (> 3000 cells/μL), (3) increased synovial polymorphonuclear neutrophil (PMN) percentage (≥ 80%), (4) more than five neutrophils per high-power field (HPF) in five HPFs (× 400), and (5) a single positive culture.

Patients with chronic PJI underwent two-stage revision, except those who did not agree to the surgery and those who were in a poor overall general condition. The surgery was performed by five experienced surgeons (two arthroplasty specialists and three knee surgeons).

Cases were categorized as culture-negative when a pathogen was not confirmed in any of the joint fluids or tissues obtained during surgery or from the knee-joint aspiration performed prior to the surgery. Alternatively, cases were categorized as culture-positive when the same pathogen was confirmed in two or more samples. This study included 136 chronic PJI cases (126 patients) that satisfied the condition of the inclusion and exclusion criteria. The culture-negative PJI group included 57 cases and the culture-positive PJI group 79 cases. Patients who were diagnosed with culture-negative PJI from other hospitals but whose pathogens were detected during the first-stage of surgery in our hospital were included in the culture-positive group. In such a case, because appropriate antibiotics against the detected bacteria before the second-stage surgery were selected, patients were classified as the culture-positive group.

**Fig. 1:**
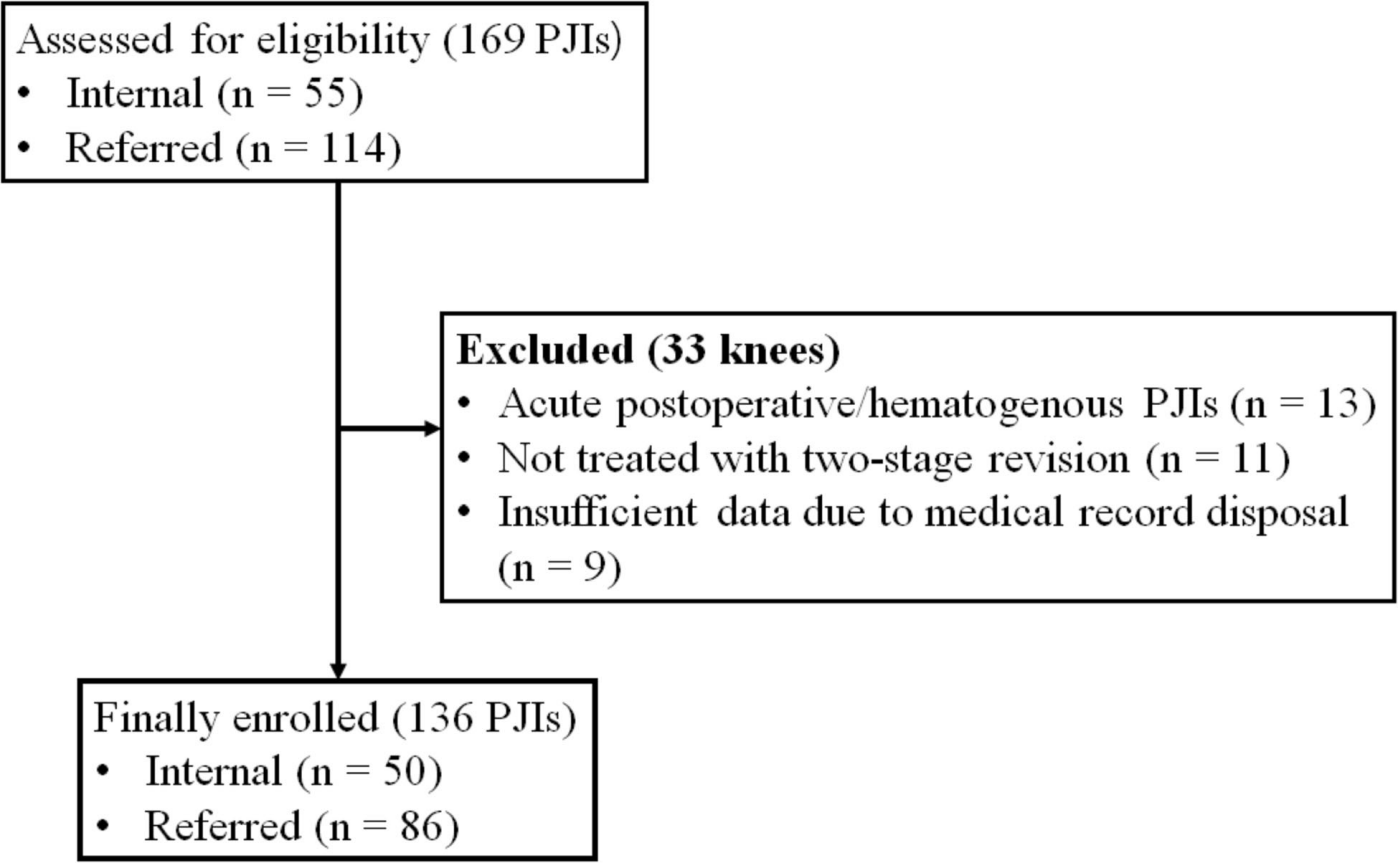
Patient flow chart

In some cases, pathogens were not confirmed at the first-stage surgery, but pathogens were later confirmed during additional surgery such as for cement spacer change. However, such a case was not included in our final study subject. The patient flow chart and demographic data are summarized in Fig. 1 and Table 1, respectively.

**Table 1:** Overall demographic data

Characteristic	Culture-negative group	Culture-positive group	*p* value
Number of knees	57	79	
Sex, male:female, *n*	14:43	16:63	0.676
Age, year	72.4 ± 7.0 (59–88)	71.1 ± 7.1 (56–86)	0.375
Body mass index, kg/m^2^	25.2 ± 3.1 (20.4–32.6)	25.9 ± 2.7 (19.6–32.4)	0.175
Source of referral, internal/external, *n*	15/42	35/44	**0.047**
ASA classification, 1/2/3/4, *n*	4/34/18/1	6/47/26/0	0.845
Type of surgery Primary/revision, *n*	53/4	70/9	0.557
Sinus tract, *n*	7 (12.3%)	19 (24.1%)	0.121
Duration from the TKA and diagnosis of PJI, month	36.0 ± 44.7 (2–240)	38.8 ± 46.2 (2–216)	0.829
Follow-up duration, month	31.8 ± 22.9 (2–134)	32.9 ± 22.9 (1–120)	0.774
Prior use of antibiotics, *n*			0.111
Yes	41 (71.9%)	43 (54.4%)	
No	9 (15.8%)	22 (27.8%)	
Unknown	7 (12.3%)	14 (17.7%)	

Data are presented as mean ± standard deviation (range) unless otherwise indicated. *ASA* American Society of Anesthesiologist

### Surgical technique and interim management

The surgical technique of the two-stage revision using the cement spacer is similar to the one described in the previous study [21]. In first-stage surgery, an antibiotic-impregnated cement spacer and/or beads were inserted after the removal of the infected prosthesis and thorough debridement. Skin incision was performed over the incision of the primary TKA. Skin in the region of a fistula was elliptically removed. During surgery, three to five samples were obtained for tissue culture.

An articulating-type or static-type cement spacer was used. The articulating cement spacer was made using a mold. A 40-g-bag of polymethylmethacrylate cement powder was mixed with 2 g of vancomycin. An average of two to three bags of cement was used to create spacers for the femur and tibia. The extension gap of the knee joint was evaluated to design the spacer on the tibial side; it was made in a way to ensure that the thickness did not cause hyperextension. When the spacers on the femoral and tibial sides were completely hardened, one bag of cement was applied to the femur and tibia, and they were added to the two spacers in the late doughy stage of setting. Each spacer was placed in the femur and tibia. A static cement spacer was inserted in cases where the use of the articulating cement spacer was difficult due to severe bone defect. For the static cement spacer, a hand-molded cement block was placed between the femur and tibia, and the lower extremity alignment was maintained until the cement completely hardened.

After first-stage surgery, infection control was assessed through physical examination and laboratory tests, including ESR and CRP levels. Joint-fluid analysis to evaluate infection control was not routinely done.

Antibiotics were administered parenterally for 4–8 weeks (Table 2). Subsequently, they were either changed to orally administered antibiotics or stopped, depending on the physical examination and laboratory test results. The orally administered antibiotics were administered for 1 to 2 weeks among patients who received antibiotics intravenously for 4–6 weeks if the antibiotics were needed to be administered further based on the physical examination and laboratory tests. There were no specific indications for the administration of antibiotics orally and orally administered antibiotics were administered at the surgeons’ discretion The results of the culture tests and an opinion from an infection specialist were used to determine the type and course of the antibiotics to be administered after the surgery [5, 22].

**Table 2:** Antibiotic administration according to organisms

Organism	Antimicrobial agent
*S. aureus* coagulate-positive or coagulase-negative	
Methicillin-susceptible	Cefazolin or nafcillin or ciprofloxacin or levofloxacin
Methicillin-resistant	Vancomycin or teicoplanin
*E. coli*	Ceftazidime or cefepime plus ciprofloxacin
*Enterococcus*	Penicillin G or ampicillin or amoxicillin or vancomycin
*P. aeruginosa*	Cefepime or meropenem or ceftazidime
*S. agalactiae*	Penicillin G or ampicillin or amoxicillin
Fungus (*C. albicans*, *C. parapsilosis*)	Fluconazole or micafungin
Culture-negative	Vancomycin plus ciprofloxacin or levofloxacin or ceftriaxone or ceftazidime or cefepime

The second-stage surgery (reimplantation surgery) was considered once the laboratory test and physical examination showed no signs of infection after the suspension of parenterally or orally administered antibiotics. The knee joint was exposed using the approach similar to that of the first-stage surgery. Thorough debridement was performed after the removal of the spacer. During the surgery, five to six samples were obtained for frozen biopsy. A pathologist evaluated the WBC count per HPF. A new cement spacer was implanted if ten or fewer WBC per HPF were observed in two or more samples. Constrained-type prostheses were implanted once the pathological examination showed no infection. For prosthesis implantation, 1 g of vancomycin was mixed into one bag of cement.

### Rehabilitation

Range of motion (ROM) exercise within a tolerable level was allowed after articulating cement spacer implantation. Range of motion exercise was not allowed in patients with the static-type cement spacer. Partial weight-bearing within a tolerable range was allowed with the assistance of a hinged knee brace. Range of motion exercise using continuous passive motion began after the second-stage surgery and once weight-bearing was already allowed.

### Evaluation

Prior to surgery, the patients suspected of having PJI underwent complete blood count examination with differential, ESR, CRP level, and joint-fluid analysis and culture, blood culture, urinalysis, and urine culture. Radiological assessment included standing knee anteroposterior (AP), lateral, 45° flexion knee posteroanterior (PA), Merchant, and weight-bearing whole-leg AP radiographs.

Patient information, including the age at the time of PJI diagnosis, sex, TKA type (primary or revision), duration from the TKA implementation to PJI diagnosis, use of antibiotics before being diagnosed with PJI, American Society of Anesthesiologist (ASA) classification, culture outcome, mortality, reoperation, and reinfection was obtained from the charts. For reoperation assessment, all additional surgeries related to the PJI, such as cement spacer change or wound debridement after first-stage surgery and debridement due to wound problem or implant removal because of reinfection after the second-stage surgery, were evaluated. Reinfection was defined as deep infection that recurred after second-stage surgery. Mortality was defined as PJI-related or surgery-related death. Moreover, treatment failure was defined as the failure of reimplantation, PJI-related or surgery-related death, and PJI recurrence after second-stage surgery.

### Statistical analysis

SPSS version 25.0 (SPSS, Chicago, IL, USA) was used for the statistical analysis. Statistical significance was set at *p* < 0.05.

Demographic data, mortality, reoperation, and reinfection rates were compared between the two groups. An independent *t* test or a Mann-Whitney *U* test was used to compare continuous variables and a chi-squared test or Fisher’s exact test was used to compare categorical variables.

## Results

The causative organisms in the culture-positive group are summarized in Table 3. As causative organisms, *S. aureus* was the most common in 25 cases (31.6%), and 18 cases (22.8%) of them were methicillin-resistant. Coagulase-negative Staphylococcus was the second most common with 15 cases (19.0%), followed by *E. coli* with 8 cases (10.1%).

**Table 3:** Causative organisms in the culture-positive group

Organism	*N*	Percentage
*S. aureus*	25 (18 methicillin-resistant)	31.6% (22.8%)
Coagulase-negative Staphylococcus	15 (7 methicillin-resistant)	19.0% (8.9%)
*E. coli*	8	10.1%
Enterococcus	7	8.9%
*P. aeruginosa*	6	7.6%
*S. agalactiae*	6	7.6%
Fungus (*C. albicans*, *C. parapsilosis*)	4	5.1%
*C. minutissimum*	3	3.8%
Other organisms	5	6.3%

Reoperation, reinfection, mortality, and failure rates of culture-negative and culture-positive groups are summarized in Table 4. The reoperation rates during the period after the first-stage surgery and before the second-stage surgery were 25.3% (20/79) and 0.5% (6/57) in the culture-positive and culture-negative groups, respectively, showing a statistically significant difference (*p* = 0.045). Of the 136 PJI cases, 97 cases (71.3%) received reimplantation surgery (culture-negative group, 43 cases; culture-positive group, 54 cases). The reoperation rates after the second-stage surgery were 11.6% (5/43) and 13.0% (7/54) in the culture-negative and culture-positive groups, respectively. No significant difference between the two groups (*p* = 1.0) was noted.

**Table 4:** Clinical outcomes of the culture-negative group and the culture-positive group

	Culture-negative group (*n* = 57)	Culture-positive group (*n* = 79)	*p* value
Reoperation after first-stage surgery, *n* (%)	6 (10.5%)	20 (25.3%)	**0.045**
Failure to reimplant, *n* (%)	14 (24.6%)	25 (31.6%)	0.444
Duration from the first-stage to second-stage surgery, month	5.2 ± 4.2 (2–22)	5.9 ± 4.5 (2–24)	0.394
Reoperation^a^ after second-stage surgery, *n* (%)	5 (11.6%)	7 (13.0%)	1.0
Reinfection^b^ after second-stage surgery, *n* (%)	5 (13.9%)	6 (12.2%)	1.0
Mortality, *n* (%)	2 (3.5%)	3 (3.8%)	1.0
Failure rate^c^, *n* (%)	19 (33.3%)	31 (39.2%)	0.589

Data are presented as means ± standard deviation (range) unless otherwise indicated. ^a^Comparison of the reoperation rate evaluated among the subjects who received the second-stage surgery (culture-negative group: 43 cases, culture-positive group: 54 cases); ^b^Comparison of the reoperation rate evaluated among the subjects who were followed up for more than 1 year after the second-stage surgery (culture-negative group: 36 cases, culture-positive group: 49 cases); ^c^One of the following conditions was defined as failure: failure to reimplant, infection-related or surgery-related death, or recurrence of periprosthetic joint infection after the reimplantation surgery

Out of the patients diagnosed with chronic PJI, some had only undergone the first-stage surgery, but the second-stage surgery was not conducted to following patients: (1) Patients who were in an unstable general conditions that prevented them from having the second-stage surgery, (2) patients who rejected the second-stage surgery, (3) patients whose knee arthrodesis was conducted with the second-stage surgery. Of the total number of cases that received the second-stage surgery, 87.3% (36/43) of the culture-negative group and 90.7% (49/54) of the culture-positive group were followed up for more than 1 year. Among them, PJI recurred in five cases (13.9%) from the culture-negative group and six cases (12.2%) from the culture-positive group, showing no significant difference (*p* = 1.0). The mortality showed no significant difference between the culture-negative and culture-positive groups (*p* = 1.0). The culture-negative and culture-positive groups showed treatment failure rates of 29.8% and 36.7%, respectively. There was no statistically significant difference (*p* = 0.589).

## Discussion

The major finding of this study is that culture outcome did not affect the overall failure rate of two-stage revision in patients with chronic PJI. Although the reoperation rate after first-stage surgery was significantly higher in the culture-positive group, mortality, the number of the patients who did not undergo second-stage surgery, reoperation rate after two-stage surgery, and reinfection rate showed no significant difference between the culture-negative and culture-positive groups.

Several authors have reported good, long-term clinical outcome and survival after TKA [23–25]. However, in some cases, various complications may develop after TKA [26]. Periprosthetic joint infection is one of the most devastating complications and is reported to be the main reason of early failure after TKA [1–3]. Diagnosing PJI is challenging because the causative agents are not always isolated from the joint fluid or tissue obtained before or during the surgery [6, 27, 28]. Some studies have reported that the culture study reports were negative in more than 40% of patients with PJI [4, 5].

Culture-negative PJI can be caused by several factors, including low-virulence-organism infection and the use of inappropriate antibiotics. Malekzadeh et al. [29] noted that prior antimicrobial therapy is a risk factor for culture-negative PJI. In this study, the number of patients who were administered with antibiotics orally or parenterally before the PJI diagnosis was higher in the culture-negative group (70.2%) than in the culture-positive group (55.7%). However, the result was not statistically significant. Among the patients included in this study, 63.2% were referred from other institutions or hospitals. Among them, information regarding previous antibiotic treatment prior to two-stage revision could not be obtained. As a result, it was difficult to determine whether the prior use of antimicrobial therapy affected the culture outcome. Nevertheless, this study partially implies a potential correlation between the prior use of antimicrobial therapy and culture outcome.

In addition to thorough debridement of the infected tissue, the use of antibiotics that have sensitivity to the causative organism is critical for successful treatment of PJI. Whether or not the causative organisms identified in a culture can be a factor that can affect outcome and whether the outcome of culture-negative PJI is worse than that of culture-positive PJI remains unclear. Many authors have reported that culture-negative PJI and culture-positive PJI showed no considerable difference with respect to treatment success and infection control rates [5, 13–18]. However, Choi et al. [20] compared 40 cases of culture-negative PJI and 135 cases of culture-positive PJI; they reported that the failure rate was significantly lower in the culture-negative group than in the culture-positive group. However, Tan et al. [19] reported that culture-negative PJI is associated with poor outcomes and a high rate of salvage procedure necessity. The existing studies have limitations such as small sample size and inconsistent types of PJI and surgery among the studies; this warrants further research. Moreover, we tried to evaluate the outcomes between the culture-negative and culture-positive groups by restricting the research subjects to the following three conditions: TKA, chronic PJI, and two-stage revision arthroplasty.

In this study, the culture-positive group showed a higher reoperation rate after the first-stage surgery. However, the two groups showed no significant difference in other parameters including the number of the patients who did not undergo second-stage surgery, reinfection rate, and mortality. The reason for the higher reoperation rate in the culture-positive group after first-stage surgery is unknown. Despite showing no statistical difference, the fact that the number of cases having a sinus tract was slightly higher in the culture-positive group can be considered a potential reason for the difference in the reoperation rate between the groups after the first-stage surgery. Furthermore, strains of high virulence, such as antibiotic-resistant bacteria or fungi, could also have affected the increased frequency of repeated debridement in the culture-positive group. However, because of a small sample size, factors that can affect the reoperation rate after the one-stage surgery could not be evaluated.

In many studies, the success rate of the two-stage revision in PJI has been reported to be between 80 and 90% [21, 30–32]. However, studies have varying definitions of success or failure of the two-stage revision. The success or failure rate may change if the evaluated outcome of the two-stage revision included the patients who could not receive the reimplantation surgery. Gomez et al. [33] reported an 81.4% success rate for the two-stage revision in PJI cases; however, in their study, the reimplantation surgery could not be performed on 27.3% of the 504 PJI cases that received the first-stage surgery (resection arthroplasty and spacer insertion). If the recurrence of PJI is considered as a treatment failure, the failure rate of the patients who received the reimplantation surgery in our study can be as low as about 10% in both groups. However, if mortality and failure to reimplant are also included in the definition of treatment failure, the failure rate increases to 29.8% and 36.7% in the culture-negative and culture-positive groups, respectively. The two groups did not show a significant difference regardless of the definition of the treatment success, indicating that even if the culture outcome is negative, infection can be controlled by thorough debridement, implementation of two-stage revision, and use of broad-spectrum antibiotics.

This study included the following limitations. First, this study is a retrospective study and included a small sample size. Second, more than 60% of the research subjects received primary TKA in other institutions or hospitals. The proportion of the patients referred from other institution or hospital was higher in the culture-positive group. Third, data regarding the prior use of antibiotics before the diagnosis of PJI could not be obtained from some of the patients.

## Conclusions

The culture outcome had no significant effect on the outcome of the two-stage revision in patients with chronic PJI. Although the reoperation rate after first-stage surgery was significantly higher in the culture-positive group, the overall failure rate showed no significant difference between the culture-positive and culture-negative groups. A negative culture might be a good prognostic factor for chronic PJI.

## Data Availability

Not applicable

## Abbreviations

ASA: American society of anesthesiologist
CRP: C-reactive protein
ESR: Erythrocyte sedimentation rate
HPF: High-power field
MSIS: Musculoskeletal Infection Society
PA: Posteroanterior
PJI: Periprosthetic joint infection
ROM: Range of motion
TKA: Total knee arthroplasty
WBC: White blood cell
